# New York Academy of Medicine

**Published:** 1898-03

**Authors:** 


### NEW YORK ACADEMY OF MEDICINE.

### Section in Orthopaedic Surgery—Meeting of December 17th, 1897.

Dr. A. M. Phelps read a paper entitled, "A Consideration of Some of the Pathological and Mechanical Problems of Hip Disease." He presented the view that nature attempted to repair the lesion producing hip disease by inflammatory action which was a normal process of repair until the inoculation of germ life which marked the beginning of disease in the area of inflammation. The absence of inoculation gave rise to ephemeral cases of hip disease which rapidly recovered without deformity or disability, but inoculation gave rise to the ordinary type of the disease. If the phagocytes were weakened by the strumous condition of the patient, they failed to destroy the germs. If, however, germ life was destroyed, repair went on and the parts were restored to their normal condition. Cavities and foci produced in the course of hip disease by the slow growth of the bacillus of tuberculosis might be inoculated by the rapidly growing pyogenic cocci when a hot and possibly painful abscess appeared and called for the knife and drainage. The adduction flexion and inward rotation attending the third stage found a mechanical explanation in the fact that when the limb passed twenty-five degrees of flexion the abductors became internal rotators, the external rotators became abductors and the tonsor vaginæ femoris became a powerful inward rotator. In the application of mechanical treatment it should be remembered that the powerful groups of muscles acting upon the thigh did not act on an axis with the shaft but nearly on a line parallel with the axis of the neck of the femur. Lateral traction, therefore, should be made in the line of the axis of the femoral neck and not of the shaft.

Dr. G. R. Elliott said that in hip disease we had a depraved process. The whole system was at a low ebb that tended to favor the development of the disease. He thought that this condition of inactivity required the use of some form of apparatus which did not, as all the instruments now in use did, subject every part of the child's body to great expense for the sake of the hip. The ideal splint of the future would not lock up so much of the body by apparatus but would fix only the diseased joint.

Dr. R. H. Sayre advocated the use of traction to fix the joint, give it physiological rest and relieve the pressure to which the diseased bone was subjected. He thought that it was difficult to apply lateral traction by a splint, but in bed lateral traction was easily applied and added to the patient's comfort. In children, however, in whom the neck was nearer in line with the shaft of the femur than in the adult, he believed that longitudinal traction was sufficient. He thought it well to apply massage to overcome the muscular atrophy of disease, but it took a great deal of care to limit the application to the sound part and not interfere with the inflamed joint.

Dr. T. H. Manley held that all pus accumulations about a joint should be evacuated early and thoroughly. He asked Dr. Phelps' opinion of the intra-articular injection of solutions of iodoform.

Dr. Phelps said that filling a joint with an insoluble compound did more harm than good. If he found a joint in which there was fluid, he evacuated it.

Dr. A. B. Judson said that the destruction of the head and acetabulum was often cited as an evidence of the bad effects of muscular contraction and of the necessity of making traction. He thought that this destruction was rather an evidence of the bad effects of the pressure made by the weight of the body, as patients with hip disease, if unmolested, were in all except the most advanced stages on their feet as much as well children. He believed that traction was the best method of promoting fixation and in painful stages it was indispensable, but that removing the weight of the body from the joint was also an indispensable part of the treatment and useful through far longer periods than traction.

Dr. T. H. Myers had made a careful study of the ephmeral cases and believed that the lesion, of whatever nature it might be, was in the bone itself. He would make a distinction between these cases and rheumatic, gonorrhœal or other affections of the joint cavity and ligaments. He could not recall any acute case of hip disease which had not been relieved by longitudinal traction alone.

Dr. R. Whitman said that the breaking down of bone appeared to be the effect of a destructive process, aggravated by the friction of the diseased surfaces upon one another, by the weight and strain of use in the attitudes of deformity and by the muscular spasm which forced the diseased parts together. The intensity of the spasm was in inverse proportion to the fixation and rest that could be assured. When the patient was recumbent the most important means of fixing the joint was traction. The ambulatory brace should remove the weight of the body from the weakened part, but it was so ineffective in fixation that its use should be combined with splinting of the joint. He had always insisted that the hip should be slightly abducted.

Dr. Phelps said that abduction should be avoided. It was one of the difficult conditions to correct in the first and second stages.

Dr. Judson said that in recovery with anchylosis abduction was desirable. It gave a fictitious length to a limb which was probably really shortened and saved the use of a high sole or reduced its height.

Dr. Sayre thought that the limb should be in as nearly normal a position as possible, neither abducted nor adducted.

Dr. H. L. Taylor thought that about five degrees of abduction would compensate for some of the shortening and make the limb more useful.

Dr. Phelps said that anchylosis was due to the severity of the inflammation, the character of the disease, the destruction of bone and contraction of cicatricial tissue about the joint. It was prevented by the use of an apparatus which seized the pelvis and fixed the joint from the commencement of the treatment until the patient was cured. The joint being thus held at perfect rest, nature went on in her effort to cure, uninterrupted by the trauma of motion. The splint was not used to overcome deformity, but merely to hold the limb in a perfectly straight position after the deformity was corrected by bed treatment.

